# Effect of Yishenjiangyafang on Plasma Metabolomics in Senile Spontaneously Hypertensive Rats

**DOI:** 10.1155/2021/8868267

**Published:** 2021-04-08

**Authors:** Lei Zhang, Jie Yu, Yingying Liu, Weixing Guo, Yunlun Li

**Affiliations:** ^1^Affiliated Hospital of Shandong University of Traditional Chinese Medicine, Shandong, Jinan 250014, China; ^2^Shandong University of Traditional Chinese Medicine, Shandong, Jinan 250355, China; ^3^Shandong Academy of Medical Sciences, Shandong, Jinan 250014, China

## Abstract

**Objectives:**

Yishenjiangyafang is a traditional Chinese medicine used to clinically treat hypertension. This study aimed to explore the effect of yishenjiangyafang on plasma metabolomics in senile spontaneously hypertensive rats (SHRs).

**Methods:**

Twelve 50-week-old SHR (6 males and 6 females) were randomly divided into two groups: a treatment group, in which rats were intragastrically administered with yishenjiangyafang (10.08 g kg^−1^·d^−1^), and a model group, in which all SHRs were administered the same volume of saline. Six age- and gender-matched Wistar–Kyoto (WKY) rats were used as the control group. Treatment was given for 6 days per week and lasted for 8 weeks. Systolic and diastolic blood pressures of the rats were measured with the noninvasive tail artery pressure measurement system. An ultraperformance liquid chromatography quadruple electrostatic field orbit (UPLC-Q-Exactive) was used to determine metabolite changes in the plasma of SHR rats before and after yishenjiangyafang treatment in the treatment group as well as in the model and control groups.

**Results:**

After yishenjiangyafang treatment, SHRs had significant lower blood pressure. Using UPLC-Q-Exactive, we identified 26 metabolic targets of yishenjiangyafang in aged SHRs and revealed that yishenjiangyafang targeted four major metabolic pathways, linoleic acid metabolism, glycerophospholipid metabolism, arginine and proline metabolism, and steroid hormone biosynthesis.

**Conclusion:**

Yishenjiangyafang decreases the blood pressure of SHRs at least in part through targeting of four major metabolic pathways. Our study illustrates mechanisms underlying the clinical application of yishenjiangyafang in the treatment of hypertensive patients.

## 1. Introduction

The prevalence of hypertension increases with age, and in aging populations worldwide, the elderly comprise a major proportion of those with hypertension [1]. Indeed, the Seventh Report of the Joint National Committee on Prevention, Detection, Evaluation, and Treatment of High Blood Pressure (JNC-7) showed that more than 60% of individuals aged over 65 developed hypertension [2]. Moreover, the Framingham Heart Study reported that those who were free of hypertension before the age of 55 have over a 90% risk of developing hypertension during their remaining life time [3]. Indeed, the severity of hypertension and the incidence of comorbidities such as stroke and coronary heart diseases are also increasing with age [4], thus presenting great financial burdens for society and families of patients and significantly affecting the quality of life of patients. Although various treatments have been developed to manage hypertensive patients, a complete understanding of the molecular basis leading to hypertension remains an active area of research in the field.

Plasma metabolites are regarded as the “biochemical phenotype” of the overall functional state of the organism and are associated with cardiovascular diseases including hypertension [5]. For example, the plasma levels of oleic acid and myoinositol are reportedly major differential metabolites between the hypertensive patients and control subjects [6]. In addition, the EPIC Potsdam study showed that C42:4 and C44:3 of serine, glycine, and acylalkyllecithin were negatively correlated with the incidence of hypertension; that is, the higher the concentration of the abovementioned metabolites in the blood of the observed subjects, the lower the probability of hypertension within 10 years [7]. In addition, several antihypertensive drugs, including amlodipine and losartan, change plasma metabolites in hypertensive patients [8]. These findings suggest that plasma metabolites are involved in the development and progression of hypertension.

Traditional Chinese medicine has long been used to treat hypertensive patients and is effective in improving patient symptoms and quality of life and protecting target organs from hypertension-induced damage, thereby reducing the occurrence and/or delaying the progression of comorbidities [9]. However, due to the complexity of the ingredients in traditional Chinese medicine, the mechanisms causing reduction of hypertension have not been understood well.

Yishenjiangyafang, a traditional Chinese medicine, has been effectively used to treat hypertension in the clinic [10]. Yishenjiangyafang has been shown to protect against hypertension-induced kidney damage by affecting RAS in hypertensive rats [11]. However, whether yishenjiangyafang affects plasma metabolites associated with hypertension is unclear. Given that the elderly are significantly affected by hypertension, in this study, we used ultra-high-performance liquid chromatography quadruple electrostatic field orbit (UPLC-Q-Exactive), a high-resolution mass spectrometry, to study the effect of yishenjiangyafang on the plasma metabolites in senile spontaneously hypertensive rats (SHRs) before and after treatment with yishenjiangyafang. Our study illustrates metabolomics-related mechanisms underlying the application of yishenjiangyafang in the clinical treatment of hypertension.

## 2. Materials and Methods

### 2.1. Animals and Grouping

Wistar–Kyoto (WKY) rats and SHRs were purchased from Beijing Weitong Lihua Experimental Animal Technology Co., Ltd. Twelve 50-week-old SHRs (6 male and 6 female; body weight 190.46 ± 8.71 g for male and 125.38 ± 6.89 g for female) were randomly divided into two groups: a treatment group (*n* = 6), hereinafter referred to as “TG,” in which all SHRs were intragastrically administered with yishenjiangyafang (10.08 g kg^−1^·d^−1^) as previously described [12], and a model group, hereinafter referred to as “MG,” in which all SHRs were administered the same volume of saline. Age-matched 6 WKY rats (3 males with a mean body weight of 285.33 ± 9.82 g and 3 females with a mean body weight of 227.82 ± 9.36 g) were included in the control group (hereinafter referred to as “CG”), in which the rats were administered with the same volume of saline.

Treatment was given for 6 days per week and lasted for 8 weeks. The blood pressure and body weight of each rat were monitored every morning. All experimental animals were caged (6 per cage) and housed in a quiet breeding room at a temperature of 17 to 25°C, a humidity of 45% to 60%, and in a 12-hour light-dark cycle and were fed with standard feed and tap water. The animal experimental protocols were approved by the Ethics Committee of the hospital, laboratory animal certificate number SCXK (Beijing) 2017- 0006.

### 2.2. Reagents

Yishenjiangyafang (504 g/L) was provided by the Affiliated Hospital of Shandong University of Traditional Chinese Medicine (China); this prescription consisted of *Astragalus membranaceus*, *Astragalus membranaceus*, *Ligustrum lucidum*, *Epimedium*, radix *Achyranthis*, mistletoe, fried jujube seed, and *Alisma alisma*. These traditional Chinese medicines were prepared in a mixture according to a ratio of 30 : 12 : 25 : 25 : 9 : 25 : 25 : 20, respectively. Acetonitrile was purchased from Thermo Fisher (USA); methanol was purchased from Merck (USA), and formic acid was purchased from American Tedia (USA).

### 2.3. Blood Pressure Measurement

Systolic and diastolic blood pressures in the tail artery were measured using the noninvasive BESN-II four-channel animal tail artery pressure measurement system (Shanghai Yuyue Medical Equipment Co., Ltd., Shanghai, China). The average value was calculated from three independent measurements per rat.

### 2.4. Plasma Metabolomics Study

1. Rats were fasted for 12 hours and anesthetized with intraperitoneally injected 0.3% sodium pentobarbital (1 mL/100 g). Blood was collected from the inferior vena cava, placed in a tube containing heparin for anticoagulation, allowed to stand for 1 h, and centrifuged at 3500 r·min^−1^ at 4°C for 10 min. The supernatant was collected and centrifuged at 10,000 rpm for 5 min at 4°C, and the plasma samples were dispensed into 1.5 mL EP tubes. After the relevant safety tests, the plasma samples were stored at −80°C for future analysis. The plasma samples were thawed and shaken at room temperature before treatment. 100 *μ*L was taken to dissolve in 200 *μ*L acetonitrile, swirled for 2 min to mix thoroughly, refrigerated at 4°C for 6 h, and centrifuged at 4°C for 10,000 r/min for 15 min. The supernatant was frozen at −20°C for future use.
2. Plasma samples were thawed at 4°C on the ice. An aliquot of the 100 *μ*l plasma sample was precipitated after adding 400 *μ*l precooled methanol, being vortexed for 1 min, and 2-chloro-L-phenylalanine (60 *μ*g/mL) was used as an internal standard. The mixture was centrifuged at 15,000 rpm for 15 min at 4°C. The supernatant (400 *μ*L) was placed in a 2-mL EP tube, dried with nitrogen, and then, redissolved by adding the initial mobile phase of 100 *μ*L. Subsequently, the supernatants were transferred to LC-MS vials before the Ultrahigh Performance Liquid Chromatography-Quadrupole Time-of-Flight Mass Spectrometry (UHPLC-QTOF/MS) analysis. To ensure data quality for metabolic profiling, pooled quality control samples were prepared by mixing 5 *μ*L supernatant from each sample.
3. The quality control (QC) samples were inserted into the UPLC-Q-ExActive MS/MS spectral analysis detection sequence, and the principal component analysis (PCA) results were analyzed to monitor the stability and reproducibility of the method from sample pretreatment to sample detection. The QC sample was the mixed sample of all plasma samples. Before the detection of a large number of plasma samples, the QC sample was tested for two consecutive times to balance the system. Next, about 6 samples to be tested were inserted at every interval and 1 QC sample was inserted for detection. PCA analysis was performed on the obtained data matrix containing QC samples to investigate the clustering effect of QC samples in order to evaluate the reliability of data quality and the repeatability of test results.
4. An UltiMate 3000 ultraperformance liquid chromatograph (UHPLC, Thermo Scientific) was used for the HPLC analysis; a chromatographic separation was performed on a Thermo-C18 column (Hypersil GOLD aQ, 100 × 2.1 mm, 1.9 *μ*m) with a flow rate of 0.30 ml/min, a column temperature of 45°C, an injector temperature of 15°C, and each injection volume of 6 *μ*L. Mobile phase A comprised water containing 0.05% formic acid, and phase B was acetonitrile containing 0.05% formic acid.
5. The mass spectrometer had positive and negative ion modes which were used for mass spectrometry analysis using the Thermo Scientific Quaternary rod-electrostatic orbit (UPLC-Q-Exactive) (Q Exactive™ hybrid quadruple-Orbitrap MS, USA). The positive and negative ion mode detection conditions were ion source HESI, capillary voltage 3500 V, capillary temperature 320°C, source temperature 350°C, sheath gas 45 arb, auxiliary gas 10 arb, MS acquisition range 80–800 m/z, resolution 70,000, and S-Lens RF Level 55. Potential biomarker ions were further subjected to MS/MS detection to automatically optimize collision energy based on ion conditions.
6. The original spectral data (“.Raw” format) were obtained by analyzing the metabolic data of rat plasma samples and then converted to the “.mzXML” format recognizable by open source data processing software (XCMS software; http://metlin.scripps.edu/download/using ProteoWizard software (http://proteowizard.sourceforge.net/). XCMS was also used for peak identification, alignment, correction, and retention time correction and, finally, to generate a three-dimensional data matrix consisting of retention time, mass-to-charge ratio, and peak intensity.
7. Microsoft Excel software was used to convert the 3D data matrix into a “.CSV” format file, and MetaboAnalys v4.0 software was used for preprocessing and multivariate analysis of metabolomics data. The MetaboAnalys v4.0 data analysis module was used to provide the *t*-test, analysis of variance (ANOVA), Principal Component Analysis (PCA), and Partial Least Squares-Discriminant Analysis (PLS-DA) to complete the data matrix integrity test, missing value estimation, data filtering, and standardization. PCA, PLS-DA, hierarchical clustering, and other pattern recognition analysis were then carried out to examine the effect of the sample classification.
8. Differential metabolites were screened step by step. The first step used an OPLS-DA-generated S-Plot to perform preliminary screening of differential variables. The second step used a Volcano Plot for differential analysis. The third step further rescreened the data based on Variable Importance for the Projection (VIP) to exclude variables with a VIP < 1. The fourth step used the combination of Fold Change (filter the parts with FC greater than 2) and the *t*-test to screen the remaining different variables for markers with significant differences.
9. Based on the metabolomics data obtained, chemical information of biomarkers was entered in the KEGG database (The Kyoto Encyclopedia of Genes and Genomes, http://www.genome.jp/). The pathway database metabolic pathway database was then selected, inputting the name or CAS number of the biomarker to obtain the relevant metabolic pathway(s), and thus, a collection of multiple sets of information resulting from the information of a single biomarker is obtained.

### 2.5. Statistical Methods

All data analyses were completed using SPSS v22.0 statistical software. All measurement data were expressed as mean ± standard deviation (SD). According to the different indicators and data types, the following statistical tests were used as needed: ANOVA, *t* test, and rank sum test. A *p* value less than 0.05 was considered statistically significant.

## 3. Results

### 3.1. Comparison of Systolic and Diastolic Blood Pressure of Rats between Groups

We first compared the blood pressure between treatment, model, and control groups. As expected, the systolic and diastolic blood pressures of rats in the treatment and model groups were significantly higher than those of the control group (*p* < 0.01). In addition, before treatment, model and treatment groups had comparable systolic and diastolic blood pressures (*p*=1.000 > 0.05). However, yishenjiangyafang significantly decreased both diastolic and systolic blood pressure compared with the model group (both *p* < 0.01). As expected, yishenjiangyafang treatment significantly decreased systolic and diastolic blood pressures (*p* < 0.01) (Table 1). These data confirm that SHRs have higher systolic and diastolic blood pressure and that yishenjiangyafang significantly decreases the blood pressure of SHRs.

### 3.2. UPLC-MS Analysis of Plasma Metabolic Profiles of Rats in Three Groups

**Figure 1:**
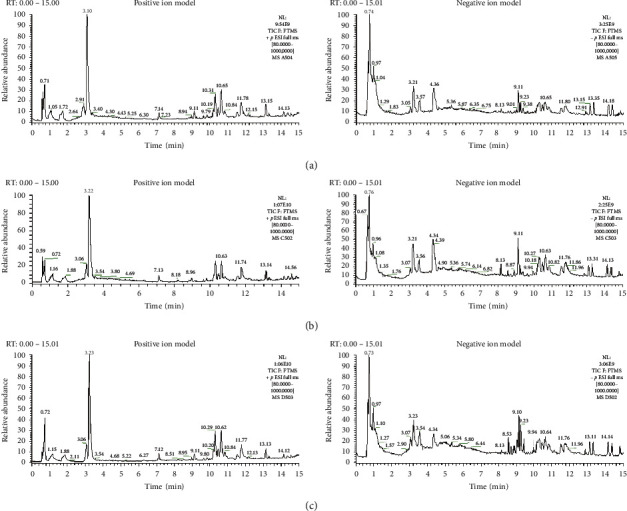
Total plasma ion chromatogram (TIC) of three groups of animals in the positive ion (a) and negative ion modes (b).

We next used UPLC-MS to analyze the metabolic profiles of the plasma samples collected from rats in the treatment, model, and control groups. The total ion chromatogram (TIC) of the two models is shown in Figure 1, i.e., the positive ion model (Figure 1(a)) and negative ion model (Figure 1(b)). While TIC showed the general common metabolic characteristics of rats from these three groups, it also revealed some differences in the peaks of these three groups. Thus, the biomarkers underlying the differences in these peaks and their biological significance need to be identified.

### 3.3. Data Processing

The obtained UPLC-MS spectra of the positive and negative ion detection modes of each group were preprocessed with R language software to obtain a two-dimensional data matrix including m/z, RT, and its peak area. A total of 1105 variables were obtained in the positive ion data, and 1026 variables were obtained from the negative data. The data normalization module of MetaboAnalyst v4.0 was then used to normalize the raw data generated by the positive and negative ion detection.

### 3.4. PCA Analysis

**Figure 2:**
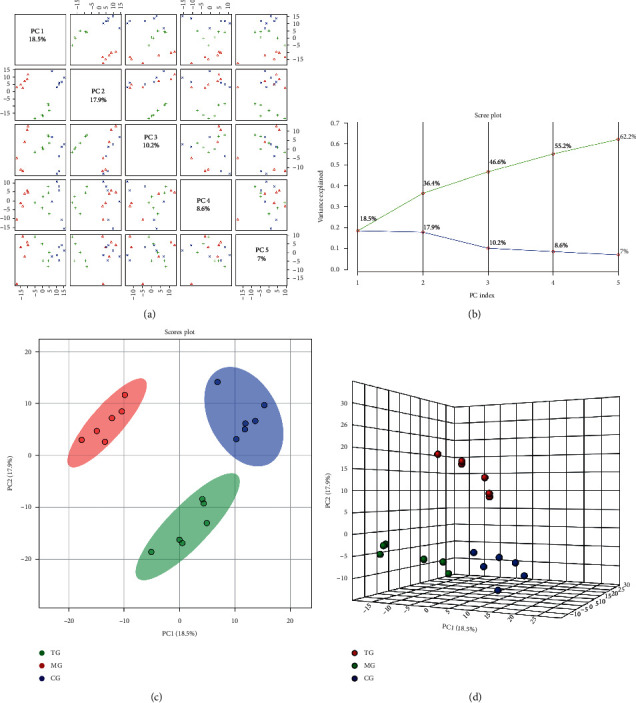
Paired scores (a), variance interpretation of gravel (b), two-dimensional score map (c), and three-dimensional score map (d) of PCA in the positive ion mode.

PCA was modeled on the standardized data matrix to observe its grouping trend. In the positive ion mode, the data matrix of each group was analyzed by unsupervised PCA (Figure 2(a) and 2(b)). A total of five principal components were obtained from the PCA analysis model, and the interpretation variances from 1 to 5 were 18.5%, 17.9%, 10.2%, 8.6%, and 7%, respectively, and the cumulative interpretation sum was 62.2%, indicating good interpretation ability. The two- and three-dimensional score maps of PCA (Figures 2(c) and 2(d)) showed that the three group samples exhibited a separation trend. As an unsupervised model, PAC has a weak ability to eliminate bias caused by intragroup differences. Hence, a supervised PLS-DA model is needed to further expand the intergroup separation.

**Figure 3:**
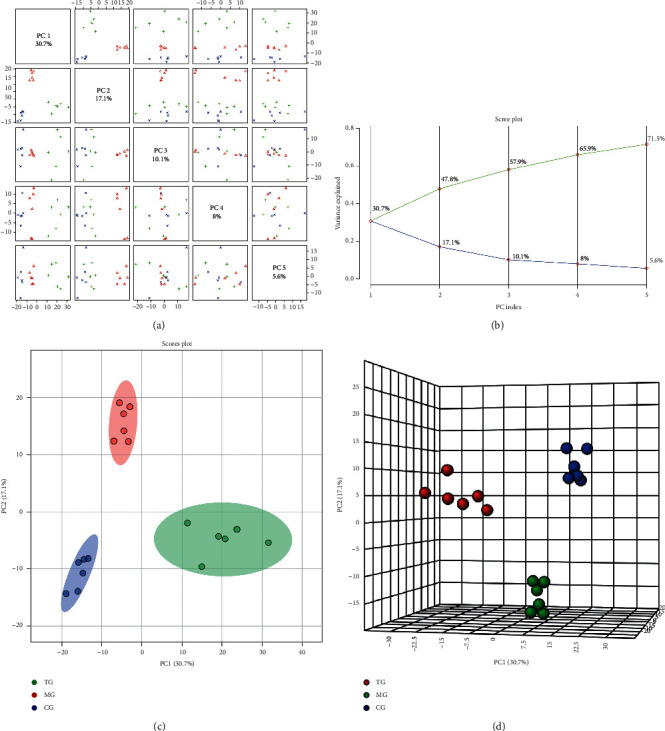
Paired scores (a), variance interpretation of gravel (b), two-dimensional score map (c), and three-dimensional score map (d) of PCA in the negative ion mode.

In the negative ion mode, the data matrix of each group was analyzed by unsupervised PCA (Figures 3(a) and 3(b)). The five principal components extracted by the PCA analysis model were interpreted by the variances of 1 to 5, respectively, 17.7%, 17.1%, 10.1%, 8%, and 5.6%, and the cumulative interpretation sum was 71.5%, indicating the good interpretation ability. Also, the two- and three-dimensional score maps obtained from PCA analysis (Figures 3(c) and 3(d)) showed that the samples from these three groups exhibited a separation trend. Thus, similar to the PCA analysis in the positive ion mode, the data separation between these groups must be further expanded by establishing a supervised PLS-DA model.

### 3.5. PLS-DA Analysis

**Figure 4:**
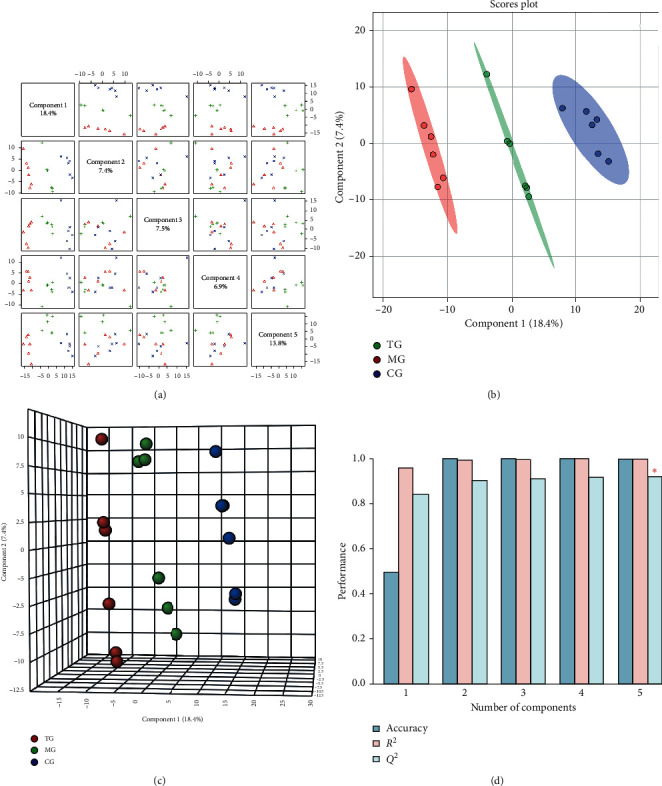
PLS-DA paired scores (a), two-dimensional score map (b), three-dimensional score map (c), and efficiency evaluation of the PLS-DA mode (d) in the positive mode.

Supervised identification was performed for the standardized data matrix of the model and the control groups, and the PLS-DA model was established to enhance the grouping and clustering effects. The positive ion detection mode (Figure 4(a)) showed that the PLS-DA analysis model generated 5 principal components, and the interpretation variances ranging from 1 to 5 were 18.4%, 7.4%, 7.5%, 6.9%, and 13.8%, respectively. PLS-DA two- and three-dimensional scores (Figures 4(b) and 4(c)) showed a significant separation between the samples from these three groups. The results of the 10-fold cross validation method (Figure 4(d), Table 2) showed that the PLS-DA model had the best effect when extracting 5 principal components with the average predictive ability *Q*^2^ of 92.07% and the prediction accuracy of 100%. These observations suggest that there may be differences in endogenous metabolic patterns between the samples from these groups.

**Figure 5:**
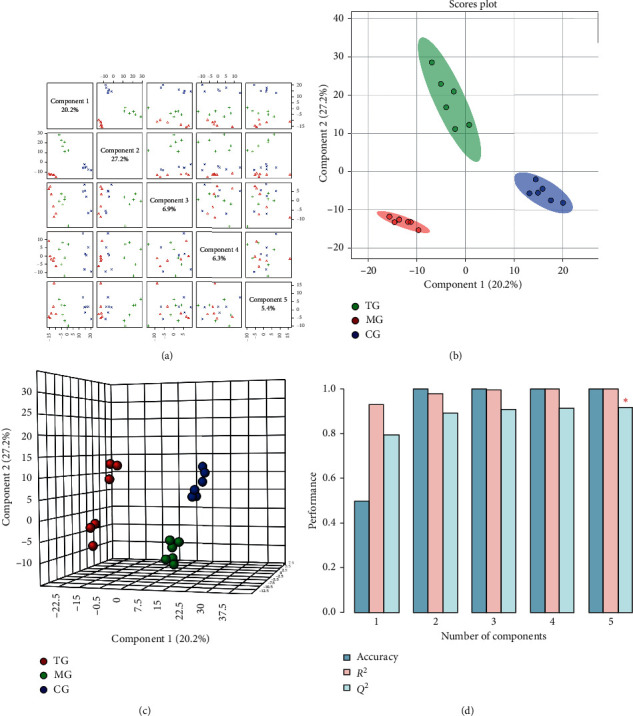
PLS-DA paired scores (a), two-dimensional score map (b), three-dimensional score map (c), and efficiency evaluation of the PLS-DA mode (d) in the negative mode.

In the negative ion mode, the PLS-DA analysis of the data matrix from each group (Figure 5(a)) showed that the first 5 interpretation variances were 20.2%, 27.2%, 6.9%, 6.3%, and 5.4%, respectively, and the PLS-DA two- and three-dimensional score maps (Figures 5(b) and 5(c)) showed that the samples from these groups exhibited significant separation. The results of the 10-fold cross validation (Figure 5(d), Table 3) showed that the PLS-DA model worked best when extracting 5 principal components with the average predictive power *Q*^2^ of 91.7% and accuracy of 100%. These findings suggest that the metabolic patterns of the three groups were different and that compared with the model group, the treatment group was closer to the control group, and the metabolic network information of the treatment group had a tendency to return to normal after yishenjiangyafang administration.

### 3.6. Hierarchical Clustering

**Figure 6:**
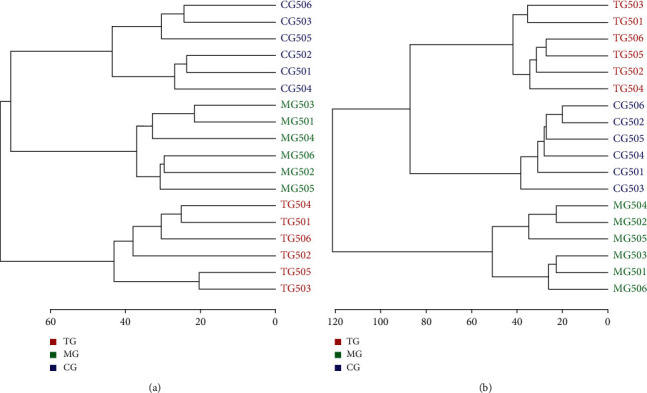
Hierarchical clustering trees of the positive (a) and negative (b) ion modes.

The hierarchical data clustering method was used to model the standardized data matrix to further investigate the grouping and clustering effects. The hierarchical clustering results of the data matrix obtained by the positive and negative ion detection modes (Figures 6(a) and 6(b)) showed that the sample of each group was clustered into one class, which showed a clear classification trend. Moreover, after treatment with yishenjiangyafang, some samples from the treatment group could be clustered with the samples from the control group into one category. Thus, the metabolic network information from the treatment group had a tendency to return to normal after yishenjiangyafang intervention of the senile SHRs.

### 3.7. Screening of Differential Biomarkers

Through the metabolite analysis of the samples from these three groups, the variables with a VIP value greater than 1 were screened by PLS-DA analysis, and the retention time and mass-to-nuclear ratio were obtained. These variables were analyzed by one-way ANOVA. The variables with a *p* value greater than 0.05 were removed, and the differential variables were used as potential biomarkers. In the positive ion mode, 56 differential metabolic markers were initially screened, while in the negative ion mode, 60 differential metabolic markers were initially screened.

### 3.8. Identification of Metabolic Markers

The differential metabolic markers obtained under the positive and negative ion models were introduced into the public biological databases such as HMDB, METLIN, and KEGG, and the related literatures were discussed. The compounds were analyzed and confirmed by two-stage MS, which finally identified 26 differential metabolic markers (Table 4).

### 3.9. Metabolic Pathway Analysis

**Figure 7:**
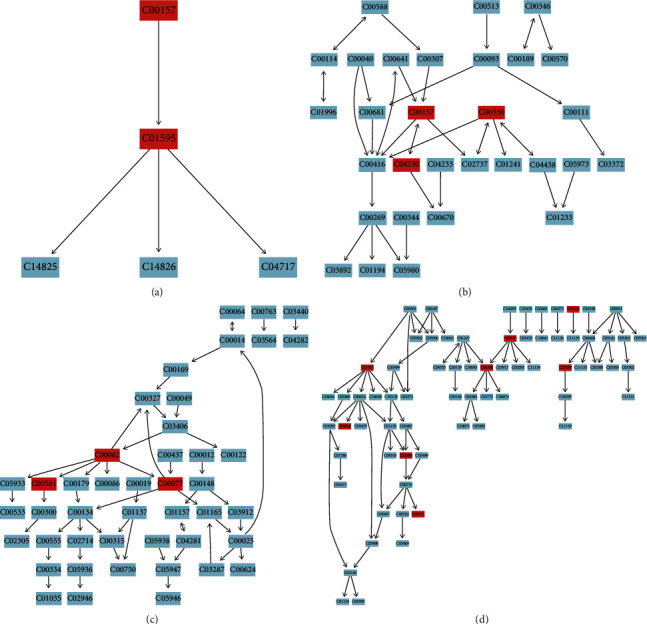
Illustrations of the linoleic acid metabolism (a), glycerophospholipid metabolism (b), arginine and proline metabolism (c), and steroid hormone biosynthesis (d).

The identified HMDB ID number of the differential metabolic markers was entered into the Pathway Analysis Module provided by MetaboAnalyst 4.0 software for metabolic pathway analysis. A metabolic pathway analysis summary table (Table 5) was generated, and the pathways with an influence value obtained from the pathway topology greater than 0.1 were analyzed as pathways for potential differential metabolic pathways. This approach identified four metabolic pathways: linoleic acid metabolism, glycerophospholipid metabolism, arginine and proline metabolism, and steroid hormone biosynthesis. Furthermore, the structural maps of each metabolic pathway were drawn (Figures 7(a)–7(d)), and the nodes in each figure were the KEGG numbers of the biomarkers, and the red biomarkers were the metabolic markers identified in the study. Among them, each metabolic pathway was a relatively independent functional module, and the red biomarker in the pathway was a key node in the functional module.

## 4. Discussion

Metabolomics is a large-scale study that analyses all low-molecular-weight metabolites in an organism or biological system during a specific physiological/pathophysiological period [13, 14]. In particular, metabolomics studies the body as a complete system and integrates whole, dynamic, comprehensive, and analytical methods. As a result, metabolomics has unique advantages for revealing the mechanisms underlying complex diseases and the metabolic mode of drugs.

In the present study, we observed that yishenjiangyafang significantly lowered the blood pressure of SHRs, as expected. We then used UPLC-Q-Exactive technology to investigate the difference in endogenous metabolic patterns between control, model, and treatment groups and identified 16 characteristic metabolic targets and four major metabolic pathways including linoleic acid metabolism, glycerophospholipid metabolism, arginine and proline metabolism, and steroid hormone biosynthesis that were specifically targeted by yishenjiangyafang.

### 4.1. Linoleic Acid Metabolism and Function

Linoleic acid is an essential fatty acid that binds to cholesterol. Deficiency in linoleic acid affects the metabolism of cholesterol, resulting in binding of cholesterol to unsaturated fatty acids and deposits on the arterial wall, ultimately leading to atherosclerosis [15]. Correspondingly, linoleic-acid-rich fats decrease the risk of developing atherosclerosis [16]. Another mechanism whereby linoleic acid protects cardiovascular systems is to generate prostaglandin E2 (PGE2) through a series of enzymatic reactions [17], and PGE2 is well known for its beneficial effects on blood pressure, water and salt metabolism, and inflammation through binding to four prostaglandin E receptors present on the cell membrane [18].

In the present study, we found that yishenjiangyafang significantly increased the plasma level of linoleic acid in elderly SHRs, which may be one of the mechanisms by which yishenjiangyafang ameliorates hypertension. It will be interesting to determine whether elderly SHRs treated with yishenjiangyafang also have increased levels of circulating PGE2.

### 4.2. Glycerophospholipid Metabolism and Function

While glycerophospholipid is a fundamental component of membranes, increased levels harm the cell membrane. For instance, glycerophospholipid-linked endothelial cell damage reduces production of nitric oxide, resulting in heightened smooth-muscle contraction and high blood pressure [19]. Phosphatidylcholine, one such glycerophospholipid, plays an important role in the metabolic process of hypertension, since phosphatidylcholine can be catalytically decomposed by different types of enzyme PLA, producing arachidonic acid and lysophosphatidylcholine, both of which mediate inflammation [20]. It should be noted that elevated inflammatory lipids such as lysophosphatidylcholine and long-chain fatty acids induce endothelial dysfunction, causing hypertension [21].

This study showed that the increased levels of phosphatidylcholine (PC), phosphatidylethanolamine (PE), and lysophosphatidylcholine (LPC) in aged SHRs were ameliorated by yishenjiangyafang, thus providing another molecular basis underlying the blood pressure decrease by yishenjiangyafang.

### 4.3. Arginine and Proline Metabolism and Function

L-arginine is a precursor of endothelial NO, and NO is an endogenous flaccid factor produced by NO synthase and exhibits potent vasodilatory and peripheral resistance-reducing activity [22]. In addition, guanidoacetic acid can be synthesized from L-arginine and is a precursor of creatine, the latter of which has a variety of physiological functions including stimulating insulin release, neuromodulation, regulating the metabolism of arginine, and mediating the oxidative state of cells [23]. In addition, ornithine is a basic amino acid which is formed by the decomposition of arginine into alkali or arginase and is associated with urea production as a part of the urea cycle [24].

In the present study, we found that yishenjiangyafang significantly increased the plasma levels of L-arginine, thioglycolic acid, and ornithine in aged SHRs. Thus, we speculate that elevated circulating levels of L-arginine and ornithine are one of the mechanisms by which Yishenjiangyafang reduces hypertension.

### 4.4. Steroid Hormone Biosynthesis

Glucocorticoid is an important hormone regulating the metabolism of a variety of substances including sugar, protein, and fat, and it plays an important role in regulating growth, development, metabolism, and the immune system. For the cardiovascular system, glucocorticoid regulates blood pressure by mediating cardiac output, the level of vascular reactivity, and the relaxation of vascular smooth muscle [25]. A previous prospective study showed that the total testosterone levels are inversely associated with the risk of hypertension in the population and that low concentrations of testosterone could serve as a biological indicator for assessing cardiovascular risk [26]. Both physiological doses of testosterone and dihydrotestosterone can upregulate the expression level of NO synthase to promote phosphorylation and increase the synthesis and release of NO in endothelial cells [27], thereby exerting its role in relaxing blood vessels and regulating intravascular balance.

We found that the levels of androsterone, pregnenolone, rostenedione, and testosterone in older SHR were significantly upregulated by yishenjiangyafang treatment, thus raising the possibility that changes in the levels of glucocorticoid are involved in yishenjiangyafang-promoted reduction in blood pressure. We noted that Yishenjiangyafang also reduced the circulating levels of corxolone and dihydrocortisol, two more forms of glucocorticoid in SHRs. However, how these changes affect the blood pressure remains to be elucidated.

## 5. Conclusions

In summary, in the present study, we used UPLC-Q-Exactive technology to obtain 26 characteristic metabolic targets of yishenjiangyafang in aged SHRs, and we finally identified four metabolic pathways that were regulated by yishenjiangyafang. Our study confirmed that Yishen Jiangyan decoction can exert antihypertensive effects by regulating linoleic acid metabolism, glycerol phospholipid metabolism, arginine and proline metabolism, steroid hormone biosynthesis, and other key metabolic targets in metabolic pathways in SHR rats. Thus, Yishen Jiangyan decoction has been demonstrated clinically for the treatment of hypertension.

Although this study initially constructed the characteristic metabolic network of SHR rats from the perspective of metabolomics, it revealed the pathophysiological mechanism of senile hypertension. However, the analysis of markers is mostly isolated, it is difficult to reflect the systemic changes of senile hypertension, and its exact mechanism needs to be further studied by the way of multiomics integration.

## Figures and Tables

**Table 1:** Comparison of blood pressure between the three groups.

Group	*n*	Systolic blood pressure		Diastolic blood pressure	
		Pretreatment	Posttreatment	Pretreatment	Posttreatment
Treatment	6	181.04 ± 3.92^△△^	170.09 ± 3.79^∗∗△△▲▲^	132.09 ± 3.33^△△^	129.00 ± 3.18^∗△△▲▲^
Model	6	181.04 ± 3.90^△△^	186.53 ± 3.90^∗△△^	132.76 ± 3.55^△△^	136.07 ± 3.92^∗△△^
Control	6	138.62 ± 3.95	138.82 ± 2.29	109.12 ± 3.19	111.18 ± 3.42

*Note.* Data are presented as mean ± SD. Intragroup comparison with pretreatment using the *t*-test, ^*∗*^*p* < 0.05, ^*∗∗*^*p* < 0.01. Intergroup comparison by the F-test, ^△△^*p* < 0.01 vs. control group; ^▲▲^*p* < 0.01 vs. model group.

**Table 2:** PLS-DA model effectiveness evaluation (positive ion mode).

Measure	1 comps	2 comps	3 comps	4 comps	5 comps
Accuracy	0	1.0	1.0	1.0	1.0
*R* ^2^	0.95956	0.99181	0.99745	0.99932	0.99977
*Q* ^2^	0.84164	0.9037	0.90976	0.91722	0.9207

**Table 3:** PLS-DA model performance evaluation (negative ion mode).

Measure	1 comps	2 comps	3 comps	4 comps	5 comps
Accuracy	0.5	1.0	1.0	1.0	1.0
*R* ^2^	0.92983	0.97839	0.99366	0.99903	0.99973
*Q* ^2^	0.79505	0.89331	0.90657	0.91366	0.917

**Table 4:** Biometabolizing marker information.

No.	m/z	Identification results	Plus-minus	Chemical formula	KEGG	HMDB	Trend
1	290.164177	Leukotriene D4	+	C_25_H_40_N_2_O_6_S	C05951	HMDB0003080	↓
2	358.274053	Pregnenolone	+	C_21_H_32_O_2_	C01953	HMDB0000253	↑
3	347.222235	2-Hydroxyestrone	+	C_18_H_22_O_3_	C05298	HMDB0000343	↑
4	799.571949	Cholic acid	+	C_24_H_40_O_5_	C00695	HMDB0000619	↓
5	464.314139	LysoPC(15:0)	+	C_23_H_48_NO_7_P	C04230	HMDB0010381	↓
6	348.228992	LysoPC(22 : 4[7Z, 10Z, 13Z, 16Z])	+	C_30_H_54_NO_7_P	C04230	HMDB0010401	↓
7	389.262502	PE(14 : 0/22:6[4Z, 7Z, 10Z, 13Z, 16Z, 19Z])	+	C_41_H_70_NO_8_P	C00350	HMDB0008847	↓
8	304.227103	Androstenedione	+	C_19_H_26_O_2_	C00280	HMDB0000053	↑
9	303.229448	Linoleic acid	+	C_18_H_32_O_2_	C01595	HMDB0000673	↑
10	185.105452	Cortexolone	+	C_21_H_30_O_4_	C05488	HMDB0000015	↓
11	406.258797	Dihydrocortisol	+	C_21_H_32_O_5_	C05471	HMDB0003259	↓
12	417.311165	Tetrahydrodeoxycorticosterone	+	C_21_H_34_O_3_	C13713	HMDB0000879	↑
13	801.585965	PC(15 : 0/16 : 1[9Z])	+	C_39_H_76_NO_8_P	C00157	HMDB0007936	↓
14	386.285977	PC(16 : 1[9Z]/16 : 1[9Z])	+	C_40_H_76_NO_8_P	C00157	HMDB0008002	↓
15	834.61196	PE(O-18 : 1[1Z]/20 : 4[5Z, 8Z, 11Z, 14Z])	+	C_43_H_78_NO_7_P	C00350	HMDB0005779	↓
16	317.247506	20a-Dihydroprogesterone	+	C_21_H_32_O_2_	C04042	HMDB0003069	↑
17	354.240345	Androsterone	+	C_19_H_30_O_2_	C00523	HMDB0000031	↑
18	143.097189	Testosterone	−	C_19_H_28_O_2_	C00535	HMDB0000234	↑
19	176.067677	Guanidoacetic acid	−	C_3_H_7_N_3_O_2_	C00581	HMDB0000128	↑
20	381.228261	Leukotriene B4	−	C_20_H_32_O_4_	C02165	HMDB0001085	↓
21	347.216075	L-arginine	−	C_6_H_14_N_4_O_2_	C00062	HMDB0000517	↑
22	427.231296	12,13-DHOME	−	C_18_H_34_O_4_	C14829	HMDB0004705	↑
23	230.064565	5-Aminopentanoic acid	−	C_5_H_11_NO_2_	C00431	HMDB0003355	↑
24	358.29628	Sphingosine	−	C_18_H_37_NO_2_	C00319	HMDB0000252	↑
25	153.064544	Ornithine	−	C_5_H_12_N_2_O_2_	C00077	HMDB0000214	↑
26	464.301762	Glycocholic acid	−	C_26_H_43_NO_6_	C01921	HMDB0000138	↓

*Note.* Plus-minus: “+” refers to the positive ion mode, “−” refers to the negative ion mode; KEGG: the identification number of a compound in the KEGG database; HMDB: the identification number of a compound in the HMDB database; trend: the trend of biomarker levels in the treatment group compared with the model group: “↑”: rising, “↓”: decreasing.

**Table 5:** Results of metabolic pathway analysis.

No.	Pathway	Total	Expected	Hits	Impact
1	Linoleic acid metabolism	5	0.12839	2	1
2	Glycerophospholipid metabolism	30	0.77033	3	0.275
3	Arginine and proline metabolism	44	1.1298	3	0.23297
4	Steroid hormone biosynthesis	70	1.7974	8	0.20944

*Note.* Total: the total number of compounds identified in the specified pathway. Hits: the exact number of matches in the uploaded marker data. Expected: the expected value of the pathway obtained by topological analysis. Impact: the influence value of the pathway obtained by topological analysis.

## Data Availability

The data used to support the findings of this study are available from the corresponding author upon reasonable request.
